# What is the size of Australia’s sexual minority population?

**DOI:** 10.1186/s13104-020-05383-w

**Published:** 2020-11-16

**Authors:** Tom Wilson, Jeromey Temple, Anthony Lyons, Fiona Shalley

**Affiliations:** 1 Melbourne School of Population and Global Health, The University of Melbourne, Melbourne, VIC Australia; 2 Australian Research Centre in Sex, Health and Society, La Trobe University, Melbourne, VIC Australia; 3 School of Psychology and Public Health, La Trobe University, Melbourne, VIC Australia; 4 Northern Institute, Charles Darwin University, Darwin, NT Australia

**Keywords:** Lesbian, Gay, Bisexual, Sexual minority, Population estimates, Australia

## Abstract

**Objectives:**

The aim is to present updated estimates of the size of Australia’s sexual minority adult population (gay, lesbian, bisexual, and other sexual minority identities). No estimate of this population is currently available from the Australian Bureau of Statistics, and very little is available from other sources. We obtained data on sexual minority identities from three data collections of two national surveys of recent years. Combining averaged prevalence rates from these surveys with official Estimated Resident Population data, we produce estimates of Australia’s sexual minority population for recent years.

**Results:**

According to percentages averaged across the three survey datasets, 3.6% of males and 3.4% of females described themselves with a minority sexual identity. When applied to Estimated Resident Populations, this gives a sexual minority population at ages 18 + in Australia of 599,500 in 2011 and 651,800 in 2016. Population estimates were also produced by sex and broad age group, revealing larger numbers and higher sexual minority percentages in the younger age groups, and smaller numbers and percentages in the oldest age group. Separate population estimates were also prepared for lesbian, gay, bisexual, and other sexual minority identities.

## Introduction

How many people in Australia identify themselves as lesbian, gay, bisexual or an alternative sexual minority orientation (e.g., queer, pansexual)? The question is difficult to answer because the Australian Bureau of Statistics (ABS) does not publish population estimates which include a sexual identity breakdown, nor does it directly collect data on sexual identity in the census or its continuous large-scale surveys which would permit such estimates to be easily calculated. The availability of population statistics from other sources is extremely limited; only a handful of academic studies have attempted to estimate the size of Australia’s sexual minority population [1–4].

Despite this paucity of data, the value of population statistics by sexual identity has been increasingly recognised in recent years [5, 6]. Population estimates on sexual minorities can provide visibility and voice to those communities. They may assist in combating misinformation and stereotypes [7]. Population numbers can inform the likely demand for specialised goods and services aimed at sexual minorities. They provide the denominators for demographic rates and indicators which enable the health and wellbeing of sexual minorities to be monitored [8]. Sexual identity population statistics should also be useful in light of legislative requirements. For example, the federal Sex Discrimination Act [9] prohibits discrimination on the basis of sexual orientation, and the Aged Care Act [10] mentions “lesbian, gay, bisexual, transgender and intersex people” as a special needs group.

This paper updates and extends the sexual minority population estimates for Australia calculated previously [3]. It reports proportions of the population identifying as a sexual minority from reliable national surveys. It then presents population estimates for the sexual minority adult population of Australia in 2011 and 2016, including by age group, and by sexual identity (gay, lesbian, bisexual, and other sexual minority identities).

## Main text

### Data sources

Data on the proportions of the population with a specific sexual identity were sourced from three data collections from two representative national household surveys, namely the General Social Survey (GSS), and waves 12 and 16 of the Household, Income And Labour Dynamics in Australia (HILDA) Survey. Other large surveys also ask about sexual identity [11–13], but were not considered for this study because they cover only part of the Australian population. The GSS was undertaken by the ABS between March and June 2014 [14] with face to face computer-assisted interviewing. It achieved a sample of about 13,000 people aged 15 years and over in households (i.e., excluding institutional accommodation). HILDA is an ongoing national longitudinal study which began data collection in 2001 [15]. About 17,000 people in households are interviewed every year using both face-to-face computer-assisted interviewing and a self-completion questionnaire (which contains the sexual identity questions). We made use of data from waves 12 and 16, conducted in 2012 and 2016 respectively, when sexual identity questions were asked.

The questions on sexual identity from the surveys are reproduced in Additional file 1: Figure S1. It is important to note that responses to these questions refer to reported sexual identity, not sexual attraction or sexual behaviour. There can be quite large variations in population numbers depending on which aspect of sexual orientation is being considered [16]. Importantly, this is reported sexual identity; people who are uncomfortable disclosing a minority sexual identity may not respond to the question or may report a different sexual identity.

The 2011 and 2016 estimated resident populations (ERPs) of Australia by sex and age group were obtained from the ABS [17]. These two years were chosen because the ERPs for these years are based on 2011 and 2016 census counts and likely to be more accurate than those for non-census years, and they are close to the reference dates of the surveys.

## Methods

Sexual identity proportions were calculated from the weighted number of adults in each sexual identity category in all three datasets. The proportions were calculated by sex for individual sexual identity categories (gay, lesbian, bisexual, and other), and for the total sexual minority population—defined as the sum of those four categories. Proportions were also calculated for the total sexual minority population by broad age group and sex. We do not present proportions by sex, age group and individual sexual identity categories as the variability around the point estimates increases significantly. The don’t know, not stated/refused responses were included in the denominators of the proportions.

Population estimates by sexual identity were calculated by taking the proportion of the population in each sexual identity category derived from the surveys and multiplying them by the published ERPs of Australia for 2011 and 2016. They were prepared in three steps. First, an estimate of the total sexual minority population aged 18 + by sex was calculated. Given a lack of information to suggest that any one survey dataset was more reliable than the others, we weighted all proportions equally. Thus, the sexual minority ($M$) population ($P$) aged 18 + by sex ($s$) was calculated as:

$${P}_{s,18+}^{M} ={ P}_{s,18+}^{ERP} \frac{1}{3}\left({p}_{s,18+}^{M,GSS}+{p}_{s,18+}^{M,HILDA-12}+{p}_{s,18+}^{M,HILDA-16}\right),$$

where $ERP$ is the official estimated resident population, $p$ denotes the proportion of the population, and $GSS$, $HILDA-12$, and $HILDA-16$ refer to the three survey datasets with the HILDA labels including the survey wave number.

Second, estimates of the total sexual minority population by age groups 18–24, 25–34, 35–44, 45–54, 55–64 and 65 + were calculated. Preliminary ($pr$) estimates were calculated as:

$${P}_{s,a}^{M}\left[pr\right]={ P}_{s,a}^{ERP} \frac{1}{3}\left({p}_{s,a}^{M,GSS}+{p}_{s,a}^{M,HILDA-12}+{p}_{s,a}^{M,HILDA-16}\right),$$

where $a$ refers to age group. Then a small constraining adjustment was made to ensure these age-specific estimates summed to the overall 18 + estimate:

$${P}_{s,a}^{M} ={P}_{s,a}^{M}\left[pr\right] \frac{{P}_{s,18+}^{M}}{\sum_{a}{P}_{s,a}^{M}\left[pr\right]}$$

Third, estimates of the 18 + population by sex by individual sexual identity category were calculated:

$${P}_{s,18+}^{m}\left[pr\right]={ P}_{s,18+}^{ERP} \frac{1}{3}\left({p}_{s,18+}^{m,GSS}+{p}_{s,18+}^{m,HILDA-12}+{p}_{s,18+}^{m,HILDA-16}\right),$$

where $m$ refers to gay/lesbian, bisexual or other. As before, a slight adjustment was required to ensure consistency with the overall sexual minority estimate:

$${P}_{s,18+}^{m} ={P}_{s,18+}^{m}\left[pr\right] \frac{{P}_{s,18+}^{M}}{\sum_{m}{P}_{s,18+}^{m}\left[pr\right]}.$$

## Results

The distribution of the adult population of Australia across sexual identity categories from the three survey datasets is shown in Table 1. The total sexual minority population varies from just under 3% according to the GSS to just over 4% in HILDA wave 16, with slightly higher percentages for females than males in the GSS but not HILDA. For males, the percentage of the population identifying as gay is higher than the bisexual percentage, while for females the bisexual percentages are higher than those for lesbian in the two HILDA datasets. Interestingly, the HILDA survey results from wave 16 indicate an increase in the share of the population identifying as a sexual minority from four years earlier. Although it is not possible to determine a trend from the limited data available, an increase over time would be consistent with recent evidence from the USA and UK [18, 19]. Complicating the analysis is the fact that the percentages for heterosexual and don’t know/not stated/refused differ noticeably between HILDA and the GSS. This may be related to differences in the survey mode and list of available responses in the three surveys. Relative standard errors for the data in this table are provided in the Additional file 1.

**Table 1:** Percentage of Australia’s adult population by sexual identity and sex

Survey	GSS	HILDA	HILDA	Average
Year of survey	2014	2012	2016	All
Ages	18 +	18 +	18 +	18 +
Females				%
Lesbian	1.31	0.97	1.00	1.09
Bisexual	1.14	1.29	1.90	1.44
Other	0.71	0.74	1.11	0.85
Total sexual minority	3.15	3.00	4.01	3.39
Heterosexual	96.02	90.53	89.47	92.01
Don’t know	0.26	0.83	1.19	0.76
Refused/not stated	0.57	5.63	5.33	3.84
Males				%
Gay	1.72	1.94	2.24	1.97
Bisexual	0.63	0.81	1.09	0.84
Other	0.44	0.89	0.89	0.74
Total sexual minority	2.79	3.63	4.21	3.56
Heterosexual	96.22	91.31	90.99	92.84
Don’t know	0.36	1.00	1.36	0.91
Refused/not stated	0.63	4.06	3.44	2.71
Total				%
Lesbian/gay	1.52	1.45	1.61	1.53
Bisexual	0.89	1.05	1.50	1.15
Other	0.56	0.81	1.00	0.79
Total sexual minority	2.97	3.31	4.11	3.46
Heterosexual	96.12	90.91	90.21	92.41
Don’t know	0.31	0.92	1.28	0.84
Refused/not stated	0.60	4.86	4.40	3.29

Percentages were calculated from weighted survey data. Relative standard errors are provided in the Additional file 1 accompanying this paper

Table 2 presents the percentage of the population identifying as a sexual minority by sex and age group. Relative standard errors are also provided in the Additional file 1. There is a strong relationship between sexual minority identity and age in the GSS results whereby percentages decline with increasing age, but the relationship is less distinct in the HILDA data, especially for males. Overall, percentages are highest in the younger 18–24 and 25–34 age groups, and lowest in the 65 + age group. Amongst those aged 18–24, the percentages reach as high as 7.5% for females and 5.7% for males, while in the 65 + age group all percentages are below the population averages for each gender.

**Table 2:** Percentage of Australia’s adult population identifying as a sexual minority by age group

Survey	GSS	HILDA	HILDA	Average
Year of survey	2014	2012	2016	All
Females				%
18–24	7.16	4.61	7.52	6.43
25–34	4.85	3.54	5.44	4.61
35–44	3.94	3.13	3.66	3.58
45–54	2.46	2.33	2.83	2.54
55–64	1.33	2.56	3.26	2.38
65 +	0.19	2.24	2.37	1.60
Males				%
18–24	2.99	4.92	5.66	4.52
25–34	4.19	5.38	6.04	5.20
35–44	3.50	2.50	3.26	3.09
45–54	2.15	2.90	3.15	2.73
55–64	1.80	3.10	3.89	2.93
65 +	1.41	3.12	3.43	2.65
Total				%
18–24	4.81	4.77	6.59	5.39
25–34	4.69	4.46	5.74	4.96
35–44	3.98	2.82	3.46	3.42
45–54	2.48	2.61	2.99	2.69
55–64	1.50	2.83	3.57	2.63
65 +	0.96	2.65	2.87	2.16

Percentages were calculated from weighted survey data. Relative standard errors are provided in the Additional file 1 accompanying this paper

Population estimates for Australia’s sexual minority populations in 2011 and 2016 are shown in Table 3. The total sexual minority population of Australia aged 18 + is estimated to have been 599,500 in 2011 with slightly fewer females (296,400) than males (303,100); by 2016 it is estimated to have grown to about 651,800 (323,500 females and 328,300 males). The population is young compared to the Australian population overall, with close to half (46%) aged 18–34. The numbers in the 65 + age group are relatively small—about 63,900 in 2011 and 76,600 in 2016. The population aged 18 + identifying as lesbian/gay is estimated to have been about 286,400 in 2016 (44% of the sexual minority population), with 215,600 as bisexual (33%), and 149,700 (23%) as other. For males, the gay population was larger than the bisexual population (182,100 and 77,900 respectively), while for females the opposite was the case (104,400 lesbian and 137,800 bisexual).

**Table 3:** The estimated sexual minority population of Australia, 2011 and 2016

	2011			2016		
	Males	Females	Total	Males	Females	Total
Total sexual minority population by age group						
18–24	51,920	67,099	119,018	54,224	70,835	125,058
25–34	84,460	71,037	155,497	94,459	81,863	176,323
35–44	49,951	60,219	110,170	51,275	62,022	113,297
45–54	42,803	40,388	83,191	44,103	42,702	86,805
55–64	37,008	30,671	67,679	39,593	34,098	73,691
65 +	36,919	26,997	63,916	44,652	31,954	76,606
Total	303,061	296,411	599,472	328,306	323,474	651,781
Sexual minority populations aged 18 +						
Lesbian/gay	168,066	95,631	263,697	182,066	104,362	286,429
Bisexual	71,886	126,244	198,130	77,874	137,770	215,645
Other	63,109	74,536	137,645	68,366	81,342	149,708
Total	303,061	296,411	599,472	328,306	323,474	651,781

Sources: Calculated from average survey percentages (Table 2) and ABS ERPs. Rows and columns may not sum exactly to totals due to rounding

## Discussion

This paper has presented new estimates of Australia’s sexual minority population based on official Estimated Resident Populations and representative surveys which collect information on sexual identity. Our study shows that Australia’s sexual minority population reached about 651,700 in 2016, representing 3.5% of the adult population, a little higher than the 3.2% estimated previously [3] due to the inclusion of HILDA wave 16 data. Equivalent percentages for other countries in recent years include 3.5% for New Zealand [20], 2.5% [21] and 2.9% for the UK [19], and 4.1% for the US [22], though these statistics are not strictly comparable due to differences in questions and survey modes. We hope that the new population estimates (Table 3) will prove useful for various policy, planning and research activities.

The sexual identity population estimates reported in this paper are probably as good as possible given the available data sources, and limitations of the population estimates are listed below. The accuracy and detail of population estimates will only be enhanced if sexual identity is included in the quinquennial census or a very large-scale national survey, such as the ABS Monthly Population Survey [23].

One of the most important findings of this study is the higher proportion of younger people reporting a minority sexual identity. This suggests that a cohort effect may be at work. Sexual identities and the willingness to disclose one’s identity can be influenced by the social attitudes and legal environment of the time when each cohort passes through their formative years. Older cohorts have spent much of their lives during a time when social acceptance was lower than today [24], and this might still influence how some of them report their identity. This cohort effect may have an important role in the proportions of people reporting a sexual minority identity in future surveys. Those young cohorts with 5–8% sexual minority identities may well maintain their identities as they get older in the future, and in the current accepting environment younger cohorts replacing them are likely to report similar, or perhaps higher, percentages. If this occurs, then Australia’s known sexual minority population will increase rapidly over the coming decades, and the estimates will need regular updating.

### Limitations

This study contains several limitations.

- We assumed that the sexual minority percentages obtained from surveys undertaken between 2012 and 2016 were valid for creating 2011 and 2016 sexual minority populations. If the trend in identifying as a sexual minority is increasing, then the 2011 population might be slightly over-estimated and the 2016 population slightly under-estimated.
- The survey data were collected using different survey modes and with slightly different wording in the sexual identity question, so they are not perfectly comparable.
- The various residual categories of don’t know, not stated, and refused need careful consideration. They vary substantially between surveys and their interpretation is not straightforward. Sexual minority percentages would be slightly higher if they were excluded from denominators.
- The scope of all surveys excluded institutional accommodation which may have led to a small amount of bias.
- Our population estimates only refer to those who reported a sexual minority identity. It is therefore a ‘revealed’ population which excludes those who do not wish to disclose their sexuality (in the survey, at least).
- Finally, the sexual minority population estimates are approximate. They are based on ABS estimated resident populations, which are good quality data, but also weighted survey data based on fairly small samples of sexual minority individuals.

## Supplementary information

**Additional file 1**. **Figure S1**: Sexual identity questions in HILDA and the GSS. **Table S1**: Relative standard errors for sexual identity percentages in Table 1. **Table S2**: Relative standard errors for the sexual minority percentages in Table 2.

## Data Availability

An Excel file of sexual minority population estimates is available from the corresponding author on request.

## Abbreviations

ABS: Australian Bureau of Statistics
ERP: Estimated resident population
GSS: General Social Survey
HILDA: Household, Income and Labour Dynamics in Australia

## References

[1] Madeddu D, Grulich A, Richters J, Ferris J, Grierson J, Smith A, Allan B, Prestage G (2006). Estimating population distribution and HIV prevalence among homosexual and bisexual men. Sex Health.

[2] Prestage G, Ferris J, Grierson J, Thorpe R, Zablotska I, Imrie J, Smith A, Grulich AE (2008). Homosexual men in Australia: population, distribution and HIV prevalence. Sex Health.

[3] Wilson T, Shalley F (2018). Estimates of Australia’s non-heterosexual population. Aust Popul Stud.

[4] Callander D, Mooney-Somers J, Keen P, Guy R, Duck T, Bavinton BR, Grulich AE, Holt M, Prestage G (2020). Australian ‘gayborhoods’ and ‘lesborhoods’: a new method for estimating the number and prevalence of adult gay men and lesbian women living in each Australian postcode. Int J Geogr Inf Sci.

[5] Office for National Statistics (ONS) Measuring sexual identity: an evaluation report. 2010. https://webarchive.nationalarchives.gov.uk/20151014015853/http://www.ons.gov.uk/ons/rel/ethnicity/measuring-sexual-identity---evaluation-report/2010/index.html. Accessed 24 Sept 2010

[6] LGBTI Health Alliance. Joint statement in support of LGBTI inclusion in the 2021 Census (2019) https://lgbtihealth.org.au/joint-statement-in-support-of-lgbti-inclusion-in-the-2021-census/. Accessed 24 Mar 2020

[7] Gates GJ (2012). LGBT identity: a demographer’s perspective. Loy LA L Rev.

[8] Perales F (2019). The health and wellbeing of Australian lesbian, gay and bisexual people: a systematic assessment using a longitudinal national sample. Aust NZ J Publ Heal.

[9] Sex Discrimination Act 1984 (Cth). https://www.legislation.gov.au/Details/C2014C00002. Accessed 5 Feb 2020

[10] Aged Care Act 1997 (Cth). https://www.legislation.gov.au/Details/C2020C00054. Accessed 25 Jan 2020

[11] Australian Longitudinal Study on Women’s Health. 2020. https://www.alswh.org.au/. Accessed 14 Apr 2020

[12] SA Health. South Australia Population Health Survey Questionnaire 2020. 2020. https://www.sahealth.sa.gov.au/wps/wcm/connect/f88b47b7-ca05-4ea4-898c-1581d47bc249/SAPHS+2020_Public+Document.pdf?MOD=AJPERES&amp;CACHEID=ROOTWORKSPACE-f88b47b7-ca05-4ea4-898c-1581d47bc249-naxHPpI. Accessed 21 Feb 2020

[13] VicHealth. VicHealth Indicators Survey 2015—Supplementary Report: Sexuality. https://www.vichealth.vic.gov.au/media-and-resources/publications/vichealth-indicators-survey-2015-supplementary-report-sexuality. Accessed 21 Feb 2020

[14] Australian Bureau of Statistics (ABS). General social survey: summary results, Australia, 2014. 2015. https://www.abs.gov.au/ausstats/abs@.nsf/mf/4159.0. Accessed 21 Feb 2020

[15] Wilkins R, Laß I, Butterworth P, Esperanza V-T (2019). The household, income and labour dynamics in Australia survey: selected findings from waves 1 to 17.

[16] Richters J, Altman D, Badcock PB, Smith AMA, de Visser RO, Grulich AE, Rissel C, Simpson JM (2014). Sexual identity, sexual attraction and sexual experience: the Second Australian Study of Health and Relationships. Sex Health.

[17] Australian Bureau of Statistics (ABS) ABS.Stat. 2020. https://stat.data.abs.gov.au/. Accessed 25 Apr 2020

[18] Newport F (2018) In U.S., Estimate of LGBT Population Rises to 4.5%. https://news.gallup.com/poll/234863/estimate-lgbt-population-rises.aspx. Accessed 10 May 2020

[19] Office for National Statistics (ONS) Sexual orientation, UK: 2018. 2020 https://www.ons.gov.uk/peoplepopulationandcommunity/culturalidentity/sexuality/bulletins/sexualidentityuk/2018. Accessed 10 May 2020

[20] Statistics New Zealand (2019) New sexual identity wellbeing data reflects diversity of New Zealanders. https://www.stats.govt.nz/news/new-sexual-identity-wellbeing-data-reflects-diversity-of-new-zealanders. Accessed 10 May 2020

[21] van Kampen SC, Lee W, Fornasiero M, Husk K (2017). The proportion of the population of England that self-identifies as lesbian, gay or bisexual: producing modelled estimates based on national social surveys. BMC Res Notes.

[22] Williams Institute (2019) Adult LGBT Population in the United States. https://williamsinstitute.law.ucla.edu/publications/adult-lgbt-pop-us/. Accessed 10 May 2020

[23] Australian Bureau of Statistics (ABS) (2020) Labour Force, Australia, May 2020. https://www.abs.gov.au/AUSSTATS/abs@.nsf/Lookup/6202.0Explanatory%20Notes1May%202020?OpenDocument. Accessed 24 Jun 2020

[24] Perales F, Campbell A (2018). Who supports equal rights for same-sex couples? Evidence from Australia. Fam Matters.

