# Supplementary material for: What is the size of Australia’s sexual minority population?
Source: BMC Res Notes. 2020 Nov 16;13:535. doi: 10.1186/s13104-020-05383-w (PMC7670686; doi:10.1186/s13104-020-05383-w)
Supplement: Supplementary file 1 — Additional file 1. Figure S1: Sexual identity questions in HILDA and the GSS. Table S1: Relative standard errors for sexual identity percentages in Table 1. Table S2: Relative standard errors for the sexual minority percentages in Table 2. [file 13104_2020_5383_MOESM1_ESM.docx]

Additional file

**Household, Income and Labour Dynamics in Australia Survey**

(self-completion questionnaire, 15 years +)

Which of the following categories best describes how you think of yourself?

• Heterosexual or Straight

• Gay or Lesbian

• Bisexual

• Other

• Unsure/Don’t know

• Prefer not to say

**General Social Survey**

(face to face computer-assisted interviewing to respondents 18 years +)

Which of the following options best describes how you think of yourself?

• Straight (heterosexual)

• Gay or Lesbian

• Bisexual

• Other

• Don’t know

**Figure S1**: Sexual identity questions in HILDA and the GSS

**Table S1**: Relative standard errors for sexual identity percentages in Table 1

| Survey | **GSS†** | **HILDA‡** | **HILDA‡** |
| --- | --- | --- | --- |
| Year of survey | 2014 | 2012 | 2016 |
| *Females* |  |  |  |
| Lesbian | 0.200 | 0.001 | 0.001 |
| Bisexual | 0.188 | 0.001 | 0.002 |
| Other | 0.333 | 0.001 | 0.002 |
| Total sexual minority | 0.108 | 0.002 | 0.003 |
| Heterosexual | 0.005 | 0.006 | 0.008 |
| Don’t know | n/a | 0.001 | 0.002 |
| Refused/not stated | 0.359 | 0.005 | 0.006 |
| *Males* |  |  |  |
| Gay | 0.189 | 0.003 | 0.004 |
| Bisexual | 0.233 | 0.001 | 0.002 |
| Other | 0.316 | 0.002 | 0.005 |
| Total sexual minority | 0.130 | 0.004 | 0.005 |
| Heterosexual | 0.004 | 0.006 | 0.007 |
| Don’t know | 0.337 | 0.002 | 0.003 |
| Refused/not stated | 0.288 | 0.005 | 0.004 |
| *Persons* |  |  |  |
| Lesbian/gay | 0.149 | 0.002 | 0.002 |
| Bisexual | 0.138 | 0.001 | 0.001 |
| Other | 0.250 | 0.001 | 0.002 |
| Total sexual minority | 0.081 | 0.002 | 0.003 |
| Heterosexual | 0.003 | 0.005 | 0.007 |
| Don’t know | 0.296 | 0.001 | 0.002 |
| Refused/not stated | 0.224 | 0.004 | 0.005 |

n/a: not available; * Relative Standard Error (RSE) proportion provided by the Australian Bureau of Statistics. Raw standard error not released; ** Linearized Standard Error proportion including adjustments for complex survey design.

**Table S2**: Relative standard errors for the sexual minority percentages in Table 2

| Survey | **GSS†** | **HILDA‡** | **HILDA‡** |
| --- | --- | --- | --- |
| Year of survey | 2014 | 2012 | 2016 |
| *Females* |  |  |  |
| 18-24 | 0.263 | 0.007 | 0.011 |
| 25-34 | 0.234 | 0.006 | 0.007 |
| 35-44 | 0.253 | 0.006 | 0.006 |
| 45-54 | 0.262 | 0.004 | 0.006 |
| 55-64 | 0.404 | 0.005 | 0.013 |
| 65+ | 0.449 | 0.005 | 0.006 |
| *Males* |  |  |  |
| 18-24 | 0.381 | 0.012 | 0.010 |
| 25-34 | 0.239 | 0.012 | 0.016 |
| 35-44 | 0.275 | 0.006 | 0.007 |
| 45-54 | 0.331 | 0.005 | 0.006 |
| 55-64 | 0.324 | 0.007 | 0.013 |
| 65+ | 0.391 | 0.007 | 0.006 |
| *Persons* |  |  |  |
| 18-24 | 0.207 | 0.007 | 0.009 |
| 25-34 | 0.177 | 0.007 | 0.009 |
| 35-44 | 0.186 | 0.004 | 0.005 |
| 45-54 | 0.185 | 0.003 | 0.004 |
| 55-64 | 0.253 | 0.004 | 0.012 |
| 65+ | 0.269 | 0.005 | 0.006 |

**†** Relative Standard Error (RSE) proportion provided by the Australian Bureau of Statistics. Raw standard error not released; **‡** Linearized Standard Error proportion including adjustments for complex survey design.
